# Risk for Tuberculosis among Children

**DOI:** 10.3201/eid1209.051606

**Published:** 2006-09

**Authors:** Hiroshi Nakaoka, Lovett Lawson, S. Bertel Squire, Brian Coulter, Pernille Ravn, Inger Brock, C. Anthony Hart, Luis E. Cuevas

**Affiliations:** * Liverpool School of Tropical Medicine, Liverpool, United Kingdom;; † Zankli Medical Centre, Abuja, Nigeria;; ‡ University Hospital, Hvidovre, Denmark;; § University of Liverpool, Liverpool, United Kingdom

**Keywords:** Tuberculosis, interferon-γ, children, Nigeria, latent infections, Quantiferon, tuberculin test, research

## Abstract

Contacts of adults with tuberculosis (TB) are at risk for infection. Tests based on interferon-γ (IFN-γ) expression in response to *Mycobacterium tuberculosis* antigens may be more sensitive than the tuberculin skin test (TST). Risk for infection was assessed by using TST and an IFN-γ-based assay (QuantiFERON Gold in Tube [QFT-IT] test) for 207 children in Nigeria in 1 of 3 groups: contact with adults with smear-positive TB, contact with adults with smear-negative TB, and controls. For these 3 groups, respectively, TST results were >10 mm for 38 (49%) of 78, 13 (16%) of 83, and 6 (13%) of 46 and QFT-IT positive for 53 (74%) of 72, 8 (10%) of 81, and 4 (10.3%) of 39 (p<0.01). Most test discrepancies were TST negative; QFT-IT positive if in contact with TB-positive persons; and TST positive, QFT-IT negative if in contact with TB-negative persons or controls. TST may underestimate risk for infection with TB in children.

## Abstract 2

Risk among children is underestimated in countries with a high incidence of this disease.

Tuberculosis (TB) is the most important infectious cause of adult deaths, and persons with acid-fast bacilli (AFB) in their sputum are the most infectious group in the community (*1**,**2*). Children exposed to adults with smear-positive pulmonary TB have a high risk for infection, and this risk increases with the degree of contact (*3**,**4*). In countries with a high incidence of TB, risk for infection among children in contact with adults with TB is 30%–50%, which is much higher than that reported by industrialized countries (*5**,**6*). However, these risk estimates were established with the tuberculin skin test (TST), which has several limitations. Children vaccinated with *Mycobacterium bovis* BCG or infected with mycobacteria other than *M*. *tuberculosis* can have false-positive TST reactions, and those with malnutrition, measles, and HIV or other infections often have false-negative reactions (*7*). In areas with a high incidence of TB, low sensitivity and specificity of the TST may result in either overestimation or underestimation of the risk for transmission.

New tests based on the expression of interferon-gamma (IFN-γ) by sensitized lymphocytes in response to specific *M*. *tuberculosis* antigens (e.g., early secretory protein 6 [ESAT-6] and culture filtrate protein 10 [CFP-10]) appear to be more specific (*8*) and sensitive (*9*) than the TST in identifying latent and active TB. Although a test with these characteristics could have enormous practical implications for improving management of children at high risk, most studies have focused on adults in countries with low incidence of TB.

This study assesses the risk for latent TB infection among young household contacts of adults with pulmonary TB in Nigeria, a country with a high incidence of TB. We compared the TST and the QuantiFERON TB Gold in Tube (QFT-IT) (Cellestis International, Carnegie, Victoria, Australia) test.

## Materials and Methods

We conducted a cross-sectional study of children in contact with adults who had pulmonary TB in Abuja, Nigeria. Incidence of TB in Nigeria is among the highest in Africa, with and estimated 293 cases/100,000 persons per year, of whom 126/100,000 are smear positive. Study children were identified by visiting the households of adults whose TB had been diagnosed at enrollment in a separate study of TB diagnosis from September 2003 to November 2004 (*10*). These index adults had undergone HIV counseling and testing and had a diagnosis of TB. Briefly, screening for HIV was done with ImmunoComb HIV1 & 2 BiSpot kits (Orgenics, Yavne, Israel) for all patients enrolled in this study. Sputum samples were collected for 2 days, and smear microscopy and BACTEC culture (Becton Dickinson, Sparks, MD, USA) were conducted by trained staff at Zankli TB Research Laboratory in Abuja. Results of smear microscopy were recorded according to the grading system of the International Union Against Tuberculosis and Lung Diseases (*11*) as –, scanty, +, ++, or +++ AFB. Since all adults enrolled in the study had positive cultures, they were classified as having smear-positive or smear-negative TB.

Home visits were conducted between March and May 2005, which was >12 weeks after diagnosis of the index case of TB. Eligible children were defined as any relative in the household <15 years of age who ate food prepared in the same cooking facilities as the index patient. During a home visit, a list of the children in the family was obtained, and <5 of these children were selected randomly to participate in the study. The parents were interviewed by using a standardized questionnaire concerning medical history, degree of contact, and characteristics of the household. Information was also obtained regarding BCG vaccination, weight, height, and clinical signs of TB. The HIV status of the children was not known because there was no medical reason for obtaining it. HIV status of the parents was assessed as part of their routine investigation for diagnosis of TB.

A separate group of children <15 years of age who were not exposed to adults with TB was selected to assess the prevalence of asymptomatic infections in the community. These control children were selected by visiting households situated at least 100 m from an index patient's household to avoid the possibility of cross-infection, maintain anonymity, and use the procedures steps as in the group not exposed to TB. Children were selected after ascertaining that adults did not have symptoms of TB.

After examination, all children were tested with the TST and QFT-IT test. The QFT-IT test uses overlapping synthetic peptides (ESAT-6 and CFT-10) that are specific for *M*. *tuberculosis*. For the TST, 10 U of purified protein derivative (Chiron Vaccines Evans, Liverpool, UK), equivalent to 5 IU tuberculin were injected by using the Mantoux method (available from http://www.immunisation.nhs.uk/files/PPD_difference.pdf) on the day of the initial visit. TST readings were obtained by using the palpation method 48–72 hours later (*12*) and were classified as negative if induration was <5 mm, intermediate if 5–9 mm, and positive if >10 mm. Children with symptoms compatible with TB were referred to the hospitals for further assessment and treatment. Parents of children with a positive TST result but normal examination results were given advice and registered for follow-up to allow monitoring of symptoms. Chemoprophylaxis is not routinely given in Nigeria.

The QFT-IT test was performed according to the manufacturer's instructions. Briefly, 1 mL of blood was drawn into vacutainer (Becton Dickinson) tubes coated with either saline (negative control tube) or ESAT-6, CFP-10, and TB 7.7 peptides (*M*. *tuberculosis–*specific antigen tubes), transported to the laboratory in Abuja 2–6 hours after collection, and incubated overnight at 37°C. The tubes were then centrifuged, and the supernatant plasma was harvested and stored at –80°C until transported to Hvidovre Hospital in Copenhagen, Denmark for IFN-γ analysis with an ELISA. Technicians performing ELISAs were unaware of clinical data, including TST status. IFN-γ detected in the saline control tube was subtracted from that in the TB antigen tube. Samples with a difference >0.35 IU/mL IFN-γ after stimulation with *M*. *tuberculosis*–specific antigens were considered positive; samples with differences <0.35 IU/mL were considered negative. Calculations were performed with software provided by the manufacturer. Samples from 33 children who had high background IFN-γ levels (control value >0.7 IU/mL) were retested. These duplicate tests provided identical results and were interpreted according to the manufacturer's guidelines.

Data were analyzed by using EpiInfo version 3.2.2 (Centers for Disease Control and Prevention, Atlanta, GA, USA). The proportion (95% confidence interval) of children with positive test results was calculated according to the smear status of the index patients. Student *t* tests were used for comparing means of continuous variables, χ^2^ tests for categorical variables, and nonparametric tests for continuous variables with skewed distributions. We analyzed our results by using a generalized estimation equation because of the likelihood of clustering of cases in families. However, the precision of the estimates did not change and they were not used.

Agreement between TST and QFT-IT test results was analyzed by using kappa statistics. Disagreement between test results was also tested according to risk for infection from index patients. Children in contact with adults with smear-negative TB and community controls were classified as being at low risk for infection. Children in contact with smear-positive adults (scanty or more AFB) were classified as high risk.

Written informed consent was obtained from parents or guardians of children. Illiterate parents were asked to provide oral consent in the presence of a witness. The study protocol was approved by the Research Ethics Committee of the Liverpool School of Tropical Medicine and the Zankli Ethical and Research Review Board in Abuja, Nigeria.

## Results

Sixty index adult case-patients living in 56 households were visited. Of these, 27 (45%) were male, 27 (45%) were smear-positive, and 33 (55%) were smear-negative. The mean duration between their initial TB diagnosis and follow-up household visits was 54 weeks (range 27–88 weeks). All smear-positive index adults had completed or were receiving treatment, 15 (56%) were co-infected with HIV, and none had died. Eighteen (55%) of the 33 adults with smear-negative TB had not started treatment, 5 (15%) had completed treatment, 4 (12%) had defaulted, and 6 (18%) had died. Thirteen (39%) were co-infected with HIV, and 10 (33%) has an unknown HIV status. According to national policy, the index case-patients who had not started treatment were registered for treatment during the course of this study.

**Figure 1:**
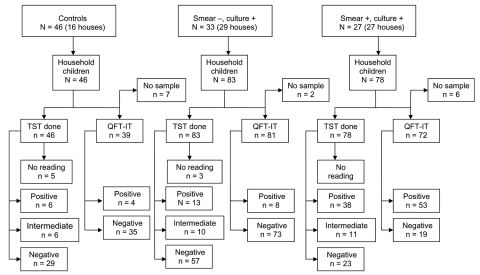
Flow chart of the study participants. TST, tuberculin skin test; QFT-IT, QuantiFERON Gold in Tube test.

Figure 1 shows a flow chart of the study participants. Of 207 children enrolled in the study, 83 were in contact with adults with smear-negative TB, 78 were in contact with adults with smear-positive TB, and 46 were community controls. A summary of their characteristics is shown in Table 1. Their mean (standard deviation) age was 7.4 (3.8) years (range 1–14 years) and 95 (46%) were male. Previous BCG vaccination was reported for 187 (90%) children, but BCG scars were present in a lower proportion of children in contact with smear-positive adults. This latter group of children also reported slightly more contact time and shared bedrooms more frequently with index-case adults than children in contact with patients with smear-negative TB (p<0.01 for both). Two (1%) children in contact with adults with smear-positive TB had cough for >3 weeks and were given therapy for TB.

**Table 1:** Characteristics of children by study group*

Characteristic	Controls (N = 46), no. (%)	Smear status of index case-patients		p value†
		Smear-negative tuberculosis (n = 83), no. (%)	Smear-positive tuberculosis (n = 78), no. (%)	
Mean (SD) age, y	6.2 (3.5)	7.5 (3.8)	7.9 (3.8)	0.04
Male/female (% male)	22/24 (48)	39/44 (47)	34/44 (44)	NS
History of *Mycobacterium bovis* BCG	42 (91)	77 (93)	68 (87)	NS
BCG scar present	18 (39)	39 (47)	19 (24)	0.01
Relationship to index case-patient				
Son or daughter	NA	47 (57)	59 (76)	<0.01
Grandchild	NA	7 (8)	0	
Sibling	NA	9 (11)	15 (19)	
Niece or nephew	NA	14 (17)	4 (5)	
Other	NA	6 (7)	0	
Mean (SD) hours in contact per day	NA	9.6 (4.9)	11.2 (5)	0.04
Shares bedroom with index case	NA	28 (34)	50 (64)	<0.01
Mean (SD) number people per bedroom	4.4 (1.8)	3.3 (1.2)	4.3 (2)	<0.01

*SD, standard deviation; NS, not significant; NA, not applicable. †Comparison of proportions and means between the 3 groups unless data do not apply to the control group.

**Figure 2:**
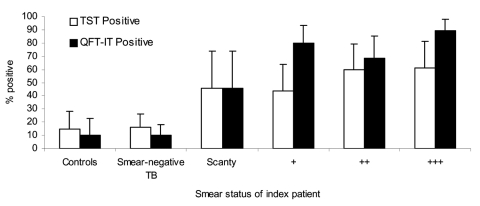
Proportion of children with positive tuberculin skin test (TST) (>10 mm) and QuantiFERON Gold in Tube (QFT-IT) test results, by adult smear positivity. Error bars show 95% confidence intervals.

TST readings were available for 193 (93%) children, and positivity varied according to the number of AFB in sputum of the adults (Figure 2). Similar proportions of control children (6/48 [15%]) and children in contact with adults with smear-negative TB (13/80 [16%]) were TST positive. A larger proportion (38/78 [53%]) of children in contact with adults with smear-positive TB were TST positive (p<0.001).

**Figure 3:**
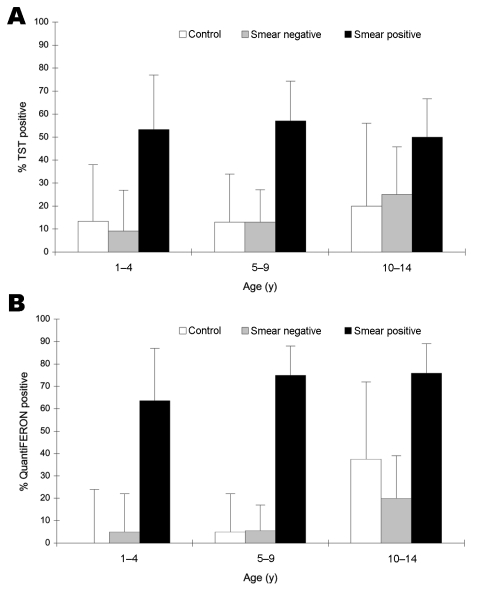
Proportion of children with positive tuberculin skin test results, by age. A) Tuberculin skin test (TST) (>10 mm). B) QuantiFERON Gold in Tube test. Error bars show 95% confidence intervals.

The relationship of the TST result with age is shown in Figure 3A. The proportion of children with positive TST results increased with age in both control children and in children in contact with adults with smear-negative TB. However, age was not a risk factor for children of adults with smear-positive TB because all children in this group had a high frequency of positive results independent of age.

QFT-IT test responses also varied according to the characteristics of the adults. Positive results were obtained for 10% (4/39) of community controls, 10% (98/81) of children in contact with adults with smear-negative TB, and 74% (53/72) of children in contact with adults with smear-positive TB (Figure 2). As was found with TST, the proportion of children with positive QFT-IT test results increased with age in community controls and in children of adults with smear-negative TB, but was high across all ages in children in contact with adults with smear-positive TB (Figure 3B).

An association was found between the QFT-IT test results and bacillary load in the sputum of the adults. Forty-five percent (4/11), 80% (16/20), 68% (15/22), and 90% (17/19) of children in contact with adults with scanty, +, ++, and +++ AFB, respectively, had positive results from the QFT-IT test (p = 0.03), as shown in Figure 2.

Comparison of test results in children with low and high risks for infection is shown in Table 2. A total of 113 children with low risk for infection and 66 with high risk for infection had both TST and QFT-IT results available. For this purpose only, children with intermediate TST responses were grouped with children with negative TST results. There was concurrence in 84 (74%) of the TST and QFT-IT test results in the low-risk group and 49 (74%) of the test results in the high-risk group (κ 0.246 and 0.498, respectively, p<0.05). In the low-risk group, most children with discordant results had positive or intermediate TST results but negative QFT-IT test results. However, in the high-risk group, most children with discordant results had negative or intermediate TST and positive QFT-IT test results.

**Table 2:** Agreement between TST and QFT-IT results in children at high risk and low risk for tuberculosis (TB)*

TST	QFT-IT	
	Positive	Negative
Low-risk group		
Positive	6	12
Intermediate	2	13
Negative	2	78
High-risk group		
Positive	34	2
Intermediate	9	0
Negative	6	15

*TST, tuberculin skin test; QFT-IT, QuantiFERON Gold in Tube test; low risk, children in contact with adults with smear-negative TB and community controls; high risk, children in contact with smear-positive adults. TST result was negative if <5 mm, intermediate if 5–9 mm, and positive if >10 mm. TST and QFN-IT test results were missing in 13 children in the low-risk group and 12 children in the high-risk group

## Discussion

TB infection often occurs in childhood, and children are often infected within the home (*5*). Children with latent TB infections have a high risk of developing overt disease and children <5 years of age are the most vulnerable. In industrialized countries, assays that measure IFN-γ expression in response to specific *M*. *tuberculosis* antigens can identify infections in exposed adults as well as in children (*13**,**14*). However, few studies have evaluated the performance of these assays in children in countries endemic for TB, and these studies have mainly focused on children with active TB (*15**,**16*). The current study assessed the risk for infection as determined by TST and QFT-IT test in children exposed to adults with microbiologically confirmed TB.

In our study, the risk for infection was largely defined by the age of the children and the smear status of the adults. In community controls, the overall rate of infection ranged from 10% (as measured by QFT-IT test) to 15% (as measured by TST) and was consistent with rates previously reported from other parts of Africa (*17**,**18*). Children in contact with adults with smear-negative TB had results similar to those of community controls, a finding consistent with the current perception that adults with smear-negative TB are less infectious (*3**,**19*). The risk for infection after exposure to adults with smear-positive TB, as determined by TST (53%), corresponds to previous values of 30% to 50% in high-incidence countries (*5**,**6*). However, the higher rate of infection determined by QFT-IT test (74%) suggests that the TST might underestimate the risk for infection for contacts of adults with smear-positive TB.

In contrast to the pattern observed for community controls, TST and QFT-IT test responses did not increase with age for contacts of smear-positive cases, suggesting that children of all ages are at high risk for infection in these households Our findings are consistent with those of previous studies of mixed-age populations that used different versions of IFN-γ tests and reported infection rates of 30% to 70% for household contacts (*16**,**20**,**21*) and an association between proximity of an index patient and positive IFN-γ responses (*13**,**14**,**20*). We also observed a trend of increasing TST and QFT-IT responders with increasing numbers of bacilli in sputum, which may indicate a dose-response relationship. This relationship has been previously reported for the TST (*5**,**20*) but is a new finding for QFT-IT test. We did not find any association between the HIV status of adults and TST and QFT-IT test results of children, but larger studies should be conducted to establish whether a relationship exists between HIV and transmission of TB within the household.

Only a few studies have assessed antigen-specific IFN-γ responses in African children with TB. Two studies found a high sensitivity (≈83%) for detection of IFN-γ in children with confirmed TB by using the ELISPOT technique (*15**,**16*). Children with active TB were more likely to have positive responses than asymptomatic children, and the assays seemed more sensitive than TST independent of age, HIV status, and malnutrition (*15*). Nicol et al. found that 50% of 26 South African children living in households with sputum-positive TB patients had TB-positive results in the IFN-γ test (*16*).

Previous studies that used with IFN-γ assays have reported a high specificity for ESAT-6 and CFP-10 (*8**,**9**,**22*). However, without a standard to determine latent TB infections and discriminate between true- and false-positive QFT-IT test results, we analyzed the agreement between the TST and QFT-IT test results to determine whether any disagreement had a different pattern in children with high and low risks for infection. In children at low risk (controls and contacts of adults with smear-negative TB), most disagreement was because TST results were positive and QFT-IT test results were negative. In contrast, in children at high risk (in contact with adults with smear-positive TB), most disagreement was because TST results were negative and QFT-IT test results were positive. This finding suggests that the pattern of disagreement was not random and may have reflected a higher sensitivity of the QFT-IT test. The correlation between TST and IFN-γ test results has been inconsistent; studies have shown good agreement in low-risk BCG unvaccinated groups (*13*) and poor agreement in BCG-vaccinated groups (*9*). These differences may reflect differences in the incidence of TB (and thus the risk for infection), laboratory methods, and cut-off values.

The advantage of new antigen-specific IFN-γ tests is their supposed high specificity. In adults, IFN-γ responses are repressed in patients with active TB (*23*) and increase after initiation of therapy for TB (*24*). In cattle, an ESAT-6-specific IFN-γ assay is reported to be a good predictor of disease severity (*25*). In humans (adults), this assay may indicate recovery from disease (*26*). It is thus plausible that the pattern of IFN-γ expression after infection is different from the pattern of TST responses over time. Thus, the former pattern might be associated with disease activity and the latter with identification of history of infection. If true, children with recent, active infections would have different IFN-γ responses than children with latent infections.

Despite increased risk of developing active TB for recent TST converters (*7**,**27*), a similar association has not yet been demonstrated for the *M*. *tuberculosis*–specific tests. Preliminary reports suggest that quantitative measurements of a patient's response may correlate with disease progression, and results from a small study in Ethiopia suggest that ESAT-6 responses might be predictive of the risk for developing active TB in subsequent months (*28*). Studies formally demonstrating such association are necessary.

A proportion of our children (≈15%, data not shown) had high IFN-γ background levels that could not be explained by technical errors. The reason for this high background may be in vivo activation of T lymphocytes specific for other infections such as malaria and other parasitic or viral diseases, which are highly prevalent in the study area. Additional studies are needed to assess the reliability of IFN-γ assays in areas with high incidence of infectious diseases. Current tests also require techniques and skills that are rarely available in the areas where most patients with TB contact the health services (*29*). Thus, more portable and simpler tests are needed to improve their applicability. Similarly, the current cost of the tests is too expensive for most developing countries (≈US $20/test). If these tests prove to be useful, marketing strategies would be needed to substantially reduce their costs.

In conclusion, exposure to adults with smear-positive TB was the most important risk factor for transmission within households. Infection rates for children exposed to adults with smear-negative TB were similar to that for community controls. The QFT-IT test detects latent TB infection more often than the TST in children of sputum-positive parents in Nigeria.
